# Natural history of cerebral visual impairment in children with cerebral palsy

**DOI:** 10.1111/dmcn.16096

**Published:** 2024-09-24

**Authors:** Jessica Galli, Erika Loi, Stefano Calza, Serena Micheletti, Anna Molinaro, Alessandra Franzoni, Andrea Rossi, Francesco Semeraro, Lotfi B. Merabet, Elisa Fazzi

**Affiliations:** ^1^ Department of Clinical and Experimental Sciences University of Brescia Brescia Italy; ^2^ Unit of Child Neurology and Psychiatry ASST Spedali Civili of Brescia Brescia Italy; ^3^ BDbiomed, BODaI Lab University of Brescia Brescia Italy; ^4^ Department of Neurological and Vision Sciences ASST Spedali Civili of Brescia Brescia Italy; ^5^ Eye Clinic, University of Brescia Brescia Italy; ^6^ The Laboratory for Visual Neuroplasticity, Department of Ophthalmology, Massachusetts Eye and Ear Harvard Medical School Boston MA USA

## Abstract

**Aim:**

To longitudinally evaluate the natural history of cerebral visual impairment (CVI) in children with cerebral palsy (CP) and identify which early visual signs or symptoms are associated with cognitive visual disorders (CVDs) at school age.

**Method:**

Fifty‐one individuals with CP and CVI underwent an ophthalmological, oculomotor, and basic visual function evaluation at three time points: T0 (6–35 months old); T1 (3–5 years old); and T2 (≥6 years old). We also performed a cognitive visual evaluation at T2. Logistic regression fitted using a generalized estimation equation (binary) and cumulative link models (ordinal) were used to model the outcomes of interest.

**Results:**

Ophthalmological deficits were stable over time, except for ocular fundus abnormalities (T1–T0, *p =* 0.01; T2–T1, *p =* 0.02; T2–T0, *p <* 0.01) and strabismus, whose frequency increased with age (T2–T0, *p*= 0.02 with T2‐T0, *p =* 0.05). Conversely, fixation (T1–T0, T2–T0, *p <* 0.01), smooth pursuit (T2–T1, T2–T0, *p <* 0.01), saccades (T1–T0, T2–T1, T2–T0, *p <* 0.01), as well as visual acuity, contrast sensitivity, and visual field (T1–T0, T2–T0, *p <* 0.01) all improved over time. Early oculomotor dysfunction was associated with CVD at T2.

**Interpretation:**

Although a diagnosis of CVI was confirmed in all children at each time point, several visual signs and symptoms improved over time; in some cases, they reached complete recovery at T1 and T2. These results emphasize the ‘permanent’ but ‘not unchanging’ nature of the CVI associated with CP during development.

## Graphical abstract

This original article is commented by Williams on pages 424–425 of this issue.

Plain language summary: https://onlinelibrary.wiley.com/doi/10.1111/dmcn.16143

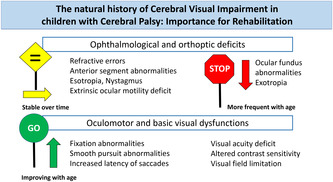

**Abbreviations**
CVD: cognitive visual disorder
CVI: cerebral visual impairment

**What this paper adds**

- Children affected by cerebral palsy often present with cerebral visual impairment, which persists during development.
- Ophthalmological and orthoptic deficits were stable over time, except for ocular fundus abnormalities and strabismus (exotropia), which were more frequent with increasing age.
- Oculomotor and basic visual dysfunctions progressively improved in preschool and school‐age children and may reach complete recovery at preschool and school age.
- Early oculomotor dysfunction is associated with cognitive visual disorders at school age.

Cerebral visual impairment (CVI) is operationally defined as a ‘verifiable visual dysfunction which cannot be attributed to disorders of the anterior visual pathways or any potentially co‐occurring ocular impairment’.^1^ Originally, the term CVI was coined to describe paediatric visual impairment of non‐ocular cause (as opposed to acquired brain injury in adults) and its association with damage to areas implicated with visual processing.^2^, ^3^ However, current views suggest that the early neurological damage associated with CVI is probably more extensive. This includes subcortical structures, optic radiations and other white matter pathways, and higher‐order visual processing areas. Thus, the term ‘cerebral visual impairment’ is considered more appropriate.^4^

CVI is the most common cause of congenital visual impairment worldwide.^4^, ^5^ It results from neurological damage, maldevelopment, or malfunctioning of retrogeniculate visual pathways and structures (i.e. optic radiations, occipital cortex, and visual associative areas).^5^ Common causes include prenatal or perinatal hypoxia‐ischaemia, head injury or trauma, infections, seizure disorder, and genetic and metabolic disorders.^5^ The clinical manifestations of CVI are heterogeneous and commonly include oculomotor disorders (such as unstable fixation, discontinuous smooth pursuit, abnormal saccadic movements, nystagmus, and strabismus), deficits in basic visual functions (e.g. reduced visual acuity and contrast sensitivity, visual field restrictions), as well as cognitive visual disorders (CVDs), including difficulties with visual recognition and visual guidance of movement.^4^, ^5^ Notably, although CVI is ascribed to dysfunction at the level of the retrogeniculate pathways, it can also co‐occur with ocular problems (e.g. ocular fundus abnormalities).^6^

To date, only a few studies have researched the natural history of CVI, focusing mainly on oculomotor and basic visual dysfunctions, such as evidence of improved smooth pursuit and saccadic eye movements,^7^ and visual acuity and contrast sensitivity with age.^8^, ^9^, ^10^, ^11^ However, several potential limitations should be considered with regard to previous studies. These include lack of a rigorous longitudinal study design, heterogeneity of several aetiologies associated with CVI, or the lack of a comprehensive evaluation of visual functions. The objective characterization of the natural history of CVI is of pivotal importance to improve counselling and the interventional strategies offered to families, and to evaluate the timing and efficacy of potential habilitative interventions.

We recently completed a cross‐sectional study analysing ophthalmological, oculomotor, and basic visual functions in 180 children diagnosed with CVI and cerebral palsy (CP) separated into three age groups (i.e. infants, preschool age children, and school‐age children).^6^ Overall, younger children showed more signs of visual impairment compared to older children. Although these findings showed different neurovisual profiles across age groups, the cross‐sectional design of the study did not allow for the evaluation of whether the signs and symptoms of CVI changed over time in the same individual.^12^ For this reason, we carried out a longitudinal study to (1) evaluate the natural history of visual functions in a sample of children with CVI and CP, detailing their neurovisual profile (including ophthalmological, oculomotor, and basic visual functions), (2) explore which early visual signs are associated with CVD later on at school age, and (3) analyse which anamnestic, clinical, and instrumental variables correlate with a better or worse neurovisual outcome. We focused this evaluation on a population of individuals diagnosed with CP, given previous evidence of a strong association of CVI with this condition (observed in approximately 70% of individuals with CP)^13^ and our ability to access a large sample of individuals with CP that could be followed longitudinally.

## METHOD

### Participants

A total of 223 children with CP were referred to our Neuro‐ophthalmological Tertiary Centre of Child Neurology and Psychiatry Unit, Civil Hospital of Brescia, Italy between May 2013 and May 2018 for suspected visual impairment. Of these, 65 children were enrolled in the study according to the following inclusion criteria: (1) a diagnosis of CP, confirmed by neurological examination and neuroradiological findings based on structural magnetic resonance imaging (MRI); (2) the presence of CVI according to previously defined criteria;^1^ and (3) aged between 6 and 35 months. The CVI diagnosis was made according to the European definition of ‘a verifiable visual dysfunction, which cannot be attributed to disorders of the anterior visual pathways or any potentially co‐occurring ocular impairment’.^1^ We enrolled all individuals with CP with a variable association of: oculomotor dysfunctions (abnormalities in fixation and/or smooth pursuit and/or saccades or abnormal ocular movements); basic visual function deficits (reduced visual acuity and/or visual field and/or altered contrast sensitivity); and larger optic disc cupping associated with optic nerve hypoplasia caused by trans‐synaptic degeneration.^14^ These visual signs were not primarily caused by disorders of the anterior visual pathways (globe, retina, or anterior optic nerve). Fourteen infants were lost to follow‐up, leading to a complete sample of 51 children (28 males, 23 females) for analysis. Individuals participating in our previous cross‐sectional study^6^ were also included in this study.

To evaluate the natural history of CVI, all participants underwent video‐recorded neurovisual evaluations at three time points (for details, see ‘Neurovisual evaluation’ section of the article): (1) T0 (6–35 months old); (2) T1 (3–5 years old); and (3) T2 (older than 6 years). Mean (SD; range) age at T0 was 23 months (8.9 months; 6–35 months); at T1, it was 55 months (11.2 months; 36–71 months); at T2, it was 96 months (20 months; 72–144 months). Forty‐four children were White (86%), three were Asian (6%), three were African or Black (6%), and one was South‐American (2%). The socioeconomic status of all families was upper‐middle; all children were recognized according to the 104/1992 Act. Table 1 reports details of the sample with regard to CP type‐based criteria as outlined in the Surveillance of Cerebral Palsy in Europe algorithm,^15^ that is, Gross Motor Function Classification System (GMFCS),^16^ Manual Ability Classification System (MACS),^17^ structural MRIs classified according to the MRI classification system proposed by the Surveillance of Cerebral Palsy in Europe,^18^ and preterm birth.

**TABLE 1:** Clinical characteristics of the study cohort.

Characteristic	*n* (%)
CP type	
Left‐sided unilateral spastic	13 (25)
Right‐sided unilateral spastic	4 (8)
Bilateral spastic	34 (67)
GMFCS level	
I	18 (35)
II	6 (12)
III	5 (10)
IV	11 (21.5)
V	11 (21.5)
MACS level	
I	20 (39)
II	9 (17.5)
III	9 (17.5)
IV	6 (12)
V	7 (14)
Structural findings	
Maldevelopment	0
Predominant white matter injury	36 (71)
Predominant grey matter injury	12 (23)
Miscellaneous	3 (6)
Preterm birth (before 37 weeks)	22 (43)
Mean (SD) gestational age, weeks	31 (3.4)
Range, weeks	24–36

Data are *n* (%) unless otherwise stated. Abbreviations: GMFCS, Gross Motor Function Classification System; MACS, Manual Ability Classification System.

The study was conducted in accordance with the ethical guidelines established by the Declaration of Helsinki and was approved by the Ethics Committee of Brescia (NP 3070). Written informed consent was obtained from all participants or a parent or caregiver before data collection.

### Neurovisual evaluation

Comprehensive video‐recorded neurovisual evaluations were conducted at the three time points according to an established protocol^6^, ^13^ (for details, see Appendix S1). Briefly, the assessment included an evaluation of ophthalmological features (i.e. cycloplegic refraction, anterior segment, and ocular fundus examination); dynamic retinoscopy and accommodation assessments were not carried out; oculomotor (i.e. visual axis alignment to detect strabismus, extrinsic ocular motility, convergence, presence or absence of nystagmus, fixation, smooth pursuit, and saccades; stereopsis was not evaluated); and basic visual functions (i.e. visual acuity, contrast sensitivity, and visual field function). When the child reached school age (T2), a cognitive visual evaluation was performed to assess visual motor and visual perceptual dysfunctions, if their IQ was determined to be normal or mildly impaired (full‐scale IQ *>* 50 and verbal IQ *>* 70 standard scores), and binocular visual acuity was not less than 3 out of 10.

### Statistical analysis

The demographic, clinical, and neuroimaging data of the sample are described as the mean, SD, and range for quantitative variables (age), and counts and percentages for qualitative variables (subtype of CP and brain MRI classification). For the description of the neurovisual profile at the three different time points, we used the mean, SD, and range for quantitative variables (visual acuity and contrast sensitivity) and the counts and percentages of impaired qualitative variables (ophthalmological, oculomotor, and visual field functions).

With regard to changes over time, we explored whether individuals underwent complete recovery of each impaired visual variable (refractive error, anterior segment, ocular fundus, strabismus, extrinsic ocular motility, nystagmus, fixation, smooth pursuit, saccades, visual acuity, contrast sensitivity, and visual field function). We also performed a comparison of neurovisual profiles between the different time points (for refraction, anterior segment, ocular fundus, strabismus, extrinsic ocular motility, nystagmus, visual acuity, contrast sensitivity, and visual field function). Binary outcomes were modelled using logistic models fitted with a generalized estimation equation^19^ to account for within‐individual measurement correlation. Results are reported as the odds ratio (OR) with 95% confidence interval (CI). Visual fixation, smooth pursuit, and saccades were treated as ordinal variables and modelled using cumulative link mixed models^20^ with ‘subject’ as the random term. Finally, we also analysed which variables among preterm birth, CP subtype (unilateral or bilateral spastic CP), severity of gross and fine motor impairment according to the GMFCS and MACS (mild, levels I and II; moderate, level III; severe, levels IV and V), neuroradiological findings, and cognitive visual evaluation (subgroup 1, children tested for CVD; subgroup 2, children not assessed), correlated with better or worse neurovisual outcome, using generalized estimation equation models. The results are reported as the estimated mean values, with corresponding 95% CIs.

Data on cognitive visual functions are described using the counts and percentages of the impaired visual motor and visual perceptual variables. The relationship between the presence of a CVD at T2 and early visual defects at T0 and T1 was evaluated using a generalized estimation equation logistic regression model. Results are reported as the OR with 95% CI.

All analyses were performed using R (R Foundation for Statistical Computing, Vienna, Austria) v4.0.3. The significance threshold was set at 5% and *p*‐values were adjusted for multiple comparisons using the Tukey algorithm.

## RESULTS

All the children presented with a variable association of more than two CVI signs and symptoms at each time point. The neurovisual profile data obtained at the three time points and from individuals with complete recovery of each visual variable are presented in Table 2.

**TABLE 2:** Neurovisual profiles at the three time points.

	T0 *n* (%)	T1 *n* (%)	T2 *n* (%)	*n* who recovered^a^ (%)	*n* who worsened^b^ (%)
Refractive errors	51 (100)	51 (100)	50 (98)	−1 (2)	0
Astigmatism (isolated and mixed)	46 (90)	45 (88)	46 (90)	/	/
Hypermetropia (isolated and mixed)	37 (72)	37 (72)	38 (74)	/	/
Myopia (isolated and mixed)	8 (16)	14 (27)	10 (20)	/	/
Anterior segment abnormalities	2 (4)	3 (6)	4 (8)	0	2 (4)
Ocular fundus abnormalities	24 (47)	31 (61)	37 (73)	0	13 (48)
Disc pallor	13 (54)	18 (58)	23 (62)	/	/
Disc cupping	5 (21)	5 (16)	6 (16)	/	/
Disc pallor and cupping	6 (25)	8 (26)	8 (22)	/	/
Strabismus	34 (67)	41 (80)	42 (82)	0	8 (47)
Esotropia	23 (68)	26 (63)	23 (55)	/	/
Exotropia	11 (32)	15 (37)	19 (45)	/	/
Extrinsic ocular motility deficit	23 (45)	25 (49)	27 (53)	0	4 (14)
Abduction deficit	12 (52)	12 (48)	12 (44)	/	/
Upshoot in adduction with ‘V' pattern	5 (21)	5 (20)	7 (26)	/	/
Downshoot or upshoot in adduction	2 (9)	2 (8)	3 (11)	/	/
Inferior oblique overaction	2 (9)	3 (12)	3 (11)	/	/
Dissociated vertical deviation	0	1 (4)	1 (4)	/	/
Convergence insufficiency	2 (9)	2 (8)	1 (4)	/	/
Nystagmus	19 (37)	20 (39)	18 (35)	−2 (10)	0
Fixation abnormalities	32 (63)	16 (31)	10 (20)	−22 (69)	0
Unstable	23 (72)	13 (81)	10 (100)	/	/
Difficult to evoke	2 (6)	0	0	/	/
Not elicited	7 (22)	3 (19)	0	/	/
Smooth pursuit abnormalities	42 (82)	38 (74)	25 (49)	−17 (40)	0
Discontinuous	32 (76)	35 (92)	25 (100)	/	/
Not elicited	10 (24)	3 (8)	0	/	/
Saccadic abnormalities	45 (88)	43 (84)	32 (63)	−13 (29)	0
Increased latency	5 (11)	7 (16)	6 (19)	/	/
Dysmetric	7 (16)	10 (23)	12 (38)	/	/
Dysmetric and with increased latency	17 (38)	20 (47)	9 (28)	/	/
Not elicited	16 (35)	6 (14)	5 (15)	/	/
Visual acuity deficit	38 (74)	28 (55)	28 (55)	−10 (26)	0
Altered contrast sensitivity	32 (63)	19 (37)	15 (29)	−17 (53)	0
Visual field limitation	35 (69)	24 (47)	17 (33)	−18 (51)	0
Right or left field defect	20 (57)	12 (50)	8 (47)	/	/
Upper or inferior field defect	1 (3)	0	0	/	/
Generalized field loss	14 (40)	12 (50)	9 (53)	/	/
CVI diagnosis	51 (100)	51 (100)	51 (100)	0 (0)	0

Abbreviation: CVI, cerebral visual impairment. 0, nobody; /, not evaluated.

^a^ Number of individuals who underwent complete recovery at T1 or T2.

^b^ Number of individuals who developed a deficit at T1 or T2.

The comparison of neurovisual profiles between T0, T1, and T2 is presented in Table 3. Regarding ophthalmological abnormalities, we noted that isolated or mixed astigmatism was the most common type of refractive error, followed by hypermetropia and myopia. No significant changes with age were detected regarding refractive errors and anterior segment anomalies. However, ocular fundus abnormalities (characterized by isolated optic disc cupping or optic disc pallor) were more frequent at preschool and school age (T1–T0, *p =* 0.01; T2–T1, *p =* 0.02; T2–T0, *p <* 0.01).

**TABLE 3:** Comparison of the neurovisual profiles between the T0, T1, and T2 time points.

	Mean score difference (95% CI)	*p*	Mean score difference (95% CI)	*p*	Mean score difference (95% CI)	*p*
	T1 vs T0		T2 vs T1		T2 vs T0	
Refractive errors						
Astigmatism	0.81 (0.23 to 2.89)	0.92	1.22 (0.34 to 4.35)	0.92	1.00 (0.19 to 5.17)	1
Hypermetropia	1.00 (0.56 to 1.76)	1	1.10 (0.59 to 2.06)	0.92	1.10 (0.54 to 2.24)	0.94
Myopia	2.03 (0.94 to 4.36)	0.07	0.64 (0.34 to 1.19)	0.21	1.31 (0.53 to 3.21)	0.75
Anterior segment abnormalities	1.53 (0.56 to 4.14)	0.57	1.36 (0.66 to 2.79)	0.57	2.08 (0.61 to 7.06)	0.33
Ocular fundus abnormalities	1.74 (1.09 to 2.77)	**0.01**	1.70 (1.04 to 2.77)	**0.02**	2.97 (1.56 to 5.65)	**< 0.01**
Strabismus	2.05 (1.12 to 3.74)	**0.01**	1.13 (0.57 to 2.24)	0.89	2.33 (1.00 to 5.39)	**0.05**
Esotropia	1.26 (0.84 to 1.90)	0.36	0.79 (0.57 to 1.07)	0.17	1.00 (0.59 to 1.69)	1
Exotropia	1.66 (0.89 to 3.07)	0.12	1.41 (0.87 to 2.28)	0.21	2.34 (1.08 to 5.08)	**0.02**
Extrinsic ocular motility deficit	1.17 (0.65 to 2.09)	0.80	1.17 (0.81 to 1.68)	0.57	1.37 (0.69 to 2.71)	0.52
Nystagmus	1.08 (0.89 to 1.31)	0.57	0.84 (0.64 to 1.11)	0.32	0.91 (0.65 to 1.29)	0.83
Visual acuity deficit	0.41 (0.21 to 0.81)	**< 0.01**	1.00 (0.69 to 1.44)	1	0.41 (0.22 to 0.76)	**< 0.01**
Altered contrast sensitivity	0.37 (0.20 to 0.69)	**< 0.01**	0.69 (0.45 to 1.05)	0.10	0.25 (0.12 to 0.53)	**< 0.01**
Visual field limitation	0.40 (0.21 to 0.77)	**< 0.01**	0.56 (0.34 to 0.90)	**0.01**	0.22 (0.11 to 0.46)	**< 0.01**

	OR (95% CI)	*p*	OR (95% CI)	*p*	OR (95% CI)	*p*
	T1 vs T0		T2 vs T1		T2 vs T0	
Fixation^a^ abnormalities	−0.68 (−0.95 to −0.41)	**< 0.01**	−0.12 (−0.31 to 0.07)	0.30	−0.80 (−1.09 to −0.50)	**< 0.01**
Smooth pursuit^b^ abnormalities	−0.05 (−0.19 to 0.08)	0.60	−0.58 (−1.00 to −0.16)	**< 0.01**	−0.63 (−1.05 to −0.22)	**< 0.01**
Saccades^c^	−0.54 (−0.93 to −0.15)	**< 0.01**	−0.93 (−1.40 to −0.47)	**< 0.01**	−1.47 (−2.01 to −0.94)	**< 0.01**
Saccadic amplitude abnormalities	−0.10 (−0.26 to 0.07)	0.35	−0.39 (−0.86 to 0.09)	0.13	−0.48 (−1.03 to 0.07)	0.10
Saccadic latency abnormalities	−0.30 (−0.55 to 0.05)	**0.01**	−0.45 (−0.73 to −0.17)	**< 0.01**	−0.75 (−1.01 to −0.49)	**< 0.01**

Bold type indicates a statistically significant *p*‐value.

^a^ Fixation was categorized using ordinal scores: 1, present; 2, mildly impaired; 3, moderately impaired; 4, severely impaired.

^b^ Smooth pursuit was categorized using ordinal scores: 1, present; 2, mildly impaired; 3, severely impaired.

^c^ Saccades were categorized using ordinal scores: 1, present; 2, normometric with increased latency; 3, dysmetric but with normal latency; 4, dysmetric with increased latency; 5, absent. The odds ratio (OR) is provided for binary outcomes (i.e. refraction, anterior segment, ocular fundus, strabismus, extrinsic ocular motility, nystagmus, visual acuity, contrast sensitivity, and visual field). For binary outcomes (such as refraction, anterior segment, etc.), estimates, confidence intervals (CIs), and *p*‐values were estimated using logistic regression fitted using a generalized estimation equation. Visual fixation, smooth pursuit, and saccades were treated as ordinal variables; thus, the mean score difference is reported. For ordinal variables, estimates, CIs, and *p*‐values were estimated using cumulative link mixed models.

Considering oculomotor dysfunctions, the frequency of strabismus increased with age (T1–T0, *p =* 0.01; T2–T0, *p =* 0.05). In particular, esotropia was observed in about half of the children at each time point, while exotropia was more frequently detected at school age (T2–T0, *p =* 0.02). A change in ocular deviation (from esotropia to exotropia or vice versa) was noted in three cases (6%). An extrinsic ocular motility deficit (mostly characterized by an abduction deficit) and nystagmus (represented by continuous jerk nystagmus) were stable over time. Conversely, visual fixation, smooth pursuit, and saccade abnormalities improved over time. Specifically, a statistically significant difference was found for fixation (T1–T0 and T2–T0, *p <* 0.01), smooth pursuit (T2–T1 and T2–T0, *p <* 0.01), and saccade (T1–T0, T2–T1, and T2–T0, *p <* 0.01) scores. Analysing the parameters of latency and amplitude of saccadic movements separately, we found that dysmetria persisted over time while increased latency decreased (T1–T0, *p =* 0.01; T2–T1 and T2–T0, *p <* 0.01).

We also observed that basic visual functions improved with age. The mean (SD) value of visual acuity at T0, T1, and T2 was 3.9 (2.9) cycles per degree (1.8 [1.7] tenths), 4.1 (3.7) cycles per degree (4.1 [3.3] tenths; logMAR equivalent for 30 children, 0.27 [0.24]), and 4.3 (3.1) cycles per degree (5.3 [3.9] tenths; logMAR equivalent for 33 children, 0.18 [0.25]). The mean value for contrast sensitivity was 37.9% (45.2%) (T0), 21.8% (35.9%) (T1), and 12.2% (25.5%) (T2). In particular, an improvement in visual acuity, contrast sensitivity, and visual field started at preschool age (T1–T0 and T2–T0, *p <* 0.01), while visual field limitations also improved at school age (T2–T1, *p =* 0.01).

Finally, 25 of 51 children (49%, 12 males and 13 females) met the criteria for the cognitive visual evaluation. The neurovisual characteristics of the subgroup of children who underwent the cognitive visual assessment (subgroup 1) and of those who were not evaluated (subgroup 2) are reported in Table S1. Briefly, the former had a milder phenotype compared to the latter, considering both CVI signs and symptoms, and IQ and GMFCS level. Impairment of cognitive visual skills was detected in 20 of 25 individuals. Specifically, three children had an isolated visual motor dysfunction (all with unilateral spastic CP and mild fine motor impairment related to one hand; MACS level between I and II), eight had an isolated visual perceptual impairment, and nine had both disorders. Five of 25 children had no evidence of CVDs. The presence of a CVD at T2 showed a statistically significant correlation with discontinuous smooth pursuit movements at T0 (*p =* 0.04). No other early visual deficits correlated with CVDs (widespread, isolated visual motor or isolated visual perceptual dysfunction) (Table S2). Isolated visual perceptual dysfunction was associated with extrinsic ocular motility deficit and increased saccadic latency (*p =* 0.05) at T1. For details, see Tables S2 to S5.

We also analysed which variables between preterm birth, CP type, gross and fine motor impairment, neuroradiological findings, and cognitive visual evaluation correlated with better or worse neurovisual outcomes. Specifically, we observed that none of the aforementioned variables were related to refractive error status, while the frequency of ocular fundus abnormalities increased, especially in children born preterm (T1–T0 and T2–T0, *p <* 0.01; T2–T1, *p =* 0.01), in children with bilateral CP (T1–T0, *p =* 0.03; T2–T0 and T2–T1, *p <* 0.01), in children with low fine (MACS levels I and II) motor impairment (T2–T0, *p <* 0.01; T2–T1, *p =* 0.03), and with predominant white matter injury (T1–T0, *p =* 0.01; T2–T0, *p <* 0.01; T2–T1, *p =* 0.01). No significant relationship with the cognitive visual evaluation was found.

Concerning oculomotor functions, the detection of strabismus increased over time in infants born preterm (T1–T0, *p =* 0.01; T2–T0, *p =* 0.02), in individuals with bilateral CP (T1–T0, *p =* 0.02), in individuals with severe gross motor impairment (T1–T0 and T2–T0, *p =* 0.03), and in individuals with predominant white matter injury (T1–T0, *p =* 0.01; T2–T0, *p =* 0.03). Conversely, an improvement in smooth pursuit and saccades with age was not related to anamnestic, clinical, or neuroradiological data, while fixation was ameliorated mainly in children not evaluated for CVD (T1–T0 and T2–T0, *p <* 0.01; T2–T0, *p =* 0.01).

Regarding basic visual functions, we observed an improvement mainly in children born at term (visual acuity: T1–T0, *p =* 0.01; T2–T0, *p <* 0.01), with bilateral CP (contrast sensitivity: T1–T0 and T2–T0, *p <* 0.01; T2–T1, *p =* 0.03), and mild fine (visual acuity and contrast sensitivity: T1–T0 and T2–T0, *p <* 0.01; visual field: T1–T0 and T2–T0, *p <* 0.01; T2–T1, *p =* 0.01) and gross motor involvement (visual acuity: T1–T0 and T2–T0, *p <* 0.01). No relationship was found with cognitive visual evaluation and the neuroradiological imaging data.

## DISCUSSION

In this longitudinal study, we examined a sample of children affected by CP and CVI by detailing their neurovisual profile during development, thus allowing a characterization of the natural history of visual function over time. At a first level, our findings are in line with the results of our previous cross‐sectional study, demonstrating an age‐related profile of visual impairments in a population of individuals with CVI.^6^

The prevalence of refractive errors, especially astigmatism, was frequent in our study sample. While the prevalence of astigmatism and hypermetropia was stable at each time point, there was an apparent trend for an increase in myopia. This finding is similar to that observed in a population of typically developing children^21^ and could be explained by intrinsic (fast progression and axial length elongation of the eye) and extrinsic (extensive near‐work for reading, studying, and school‐related demands) factors.^21^ We highly recommend that early screening and correction of refractive errors should be pursued because failure to do so can impair cognitive development,^22^ limit activities of daily living, and affect reading skills.^23^

The prevalence of ocular fundus abnormalities also worsened over time. About half of the children without anomalies during infancy (T0) presented with optic disc cupping or optic disc pallor at school age (T1). This observation may be explained by loss of the papillomacular fibres, which is associated with early brain damage.^24^ In fact, primary injury of the retrogeniculate visual pathways can secondarily affect the pregeniculate visual pathways and the retina when brain injury occurs at an early age.^25^ Awareness of the delayed onset of ocular fundus anomalies (not detected in previous examinations) may help clinicians to interpret them correctly (because of trans‐synaptic degeneration rather than an acute brain lesion), avoiding unnecessary examination and follow‐up.^26^

A similar trajectory was observed for strabismus, whose frequency increased with age. This could be because of maldevelopment of visual motor control mechanisms caused by brain damage.^27^ Specifically, none of the children in our study showed spontaneous recovery of esotropia, while about half of the sample developed exotropia at preschool and school ages. Moreover, a change in ocular deviation (from esodeviation to exodeviation or vice versa) was noted, although only in a few cases. These findings underline the instability of ocular deviation in children with CP; this is a factor that explains why none of our participants underwent surgical treatment for strabismus. Other well‐known reasons include a higher risk of overcorrection, difficulties making precise angle measurements, limited cooperation during examination, and the complex medical condition of the patient.^28^ Our data agree with previous studies reporting that even if resolution of strabismus with age is rare, conversion from esotropia to exotropia is possible;^29^ therefore, surgery in children with CP should be deferred.^30^ Conversely, other studies underline the efficacy of an early surgical approach.^31^ Finally, in the scoping review conducted by Williams et al., a variable experience was reported regarding interventions to improve ocular alignment, with inconclusive results.^32^ Early detection of strabismus and its potential complications (e.g. amblyopia, suppression, contractures, abnormal retinal correspondence) is required to avert them, with proper early treatment carried out by using glasses, eye patches, and orthoptic training, given that surgery might not necessarily prevent abnormal sensory adaptations.^33^

As in our previous work,^6^ nystagmus and extrinsic ocular motility disorders were stable with age. Regarding other oculomotor functions, we observed progressive improvement in fixation, smooth pursuit, and saccades. Specifically, fixation was more stable at preschool age and more than half of the children presenting with anomalies during infancy demonstrated spontaneous recovery at the follow‐up examination. This finding is in agreement with evidence drawn from typically developing individuals, showing that the ability to steadily fixate a target is not completely developed at birth.^34^ Rather, it develops mostly during the first 5 years of life, paralleling retinal and central nervous system maturation.^35^ Fixation is an active process that allows an observer to maintain focused attention by inhibiting potentially intrusive eye movements^36^ and is important in activities of daily living.^35^ Moreover, fixation is essential for the correct development of visual function and is a prerequisite for the development of other oculomotor functions.^35^ This may help to explain improvements in smooth pursuit and recovery occurring at school age. In fact, the development of smooth pursuit depends on the ability to fixate a stimulus at the fovea, the ability to determine target velocity on the retina, and match it with eye velocity.^35^ Our findings are supported by data from previous literature reporting that the accuracy of smooth pursuits relies on the integration of cortical and subcortical pathways and continues to improve throughout childhood and into adolescence.^36^

Although complete recovery of saccades was observed only in one‐third of the children during the follow‐up, we observed an improvement from preschool age. In particular, saccadic latency, defined as the time required to initiate an eye movement after a verbal command, was more sensitive to the effect of age than amplitude or accuracy, that is, the process of stopping the saccade in a location to optimally foveate a visual target. Previously, saccade amplitude was reported as being relatively unaffected by age.^37^ In contrast, a slow but progressive improvement in saccade latency was reported between the first months of life^38^ up to adolescence.^36^ Saccade amplitude is controlled, above all, by early developing subcortical structures of the brain and in particular the superior colliculus, which determines the position of the target and generates accurate saccadic movements towards a target location.^39^ Conversely, latency is driven by a wide range of cortical areas (such as the parietal cortex and frontal regions); thus, it is influenced by cognitive processes such as attention, working memory, long‐term memory, and decision‐making,^40^ all of which continue to develop throughout the preschool and school years. While previous reports were based largely on observations pertaining to individuals with neurotypical development, some studies hypothesized that an improvement in altered saccadic movements also occurs in individuals with CP over time (although not assessed using a longitudinal study design^6^, ^7^, ^41^).

We also observed progressive improvement in visual acuity and contrast sensitivity at preschool age, as well as visual field function at school age. Specifically, about one‐third of children with visual acuity deficits in infancy reached normal values at school age; half of the children presenting with altered contrast sensitivity and visual field limitations had a spontaneous recovery during the follow‐up. Matsuba and Jan^10^ and Watson et al.^11^ previously reported an improvement in visual acuity and contrast sensitivity in about half of their study sample over a period of several years. These apparent discrepancies may be related to methodological issues as we considered a complete recovery of functions while other authors defined improvement as a change in level of functioning. This trajectory of improvement could be related to the development of foveal cones, cortical architecture refinement, environmental factors as observed in typically developing individuals,^42^ and developmental neuroplasticity, which may mediate functional recovery of vision by activating, modulating, and strengthening residual visual signals.^43^ Regarding the recovery of visual field function, it could be related to two possibilities. First, resolution of transient dysfunction and changes in the neuronal circuits of the perilesional tissue that occur soon after brain damage.^44^ Second, the effect of an improved ability to shift attention towards a peripheral stimulus observed at school age.^45^

Finally, a large proportion (80%) of the children in our study evaluated for cognitive visual functioning presented with a CVD, with visual motor and visual perception skills often being impaired simultaneously. The presence of oculomotor dysfunctions (i.e. discontinuous smooth pursuit, altered saccadic movements, and extraocular movement deficits) at T0 and T1 were associated with a CVD at school age. Our findings are partially in line with a previous study by Morelli et al.,^46^ who reported that oculomotor dysfunctions, and visual acuity and contrast sensitivity deficits, may have a significant impact on cognitive visual functioning. A possible explanation for the apparent lack of association between CVDs and basic visual functions in our study may be related to the fact that visual acuity and contrast sensitivity were comparatively not as severely impaired in our study sample. Furthermore, only one study investigated this relationship using a longitudinal design^47^ and found a statistically significant association between oculomotor dysfunction in newborns and CVD (expressed by visual motor and visual reasoning deficits) at 5 years of age. Thus, early recognition of these risk factors for CVD could be pivotal to offer individualized habilitative interventions during a period of maximal neuroplasticity^48^ and to mitigate further emergence of cognitive visual impairment. Notably, visual motor or visual perceptual dysfunctions are typically diagnosed later in development (around 5–6 years of age) when they typically interfere with learning, social interaction, and activities of daily living.^48^

We observed that some parameters of visual outcome were related to anamnestic features (preterm or term birth), and clinical (subtype of CP and severity of motor impairment) and neuroradiological (predominant white or grey matter damage) variables, while others were not. Specifically, the frequency of ocular fundus abnormalities increased, especially in children born preterm with bilateral CP and low fine motor impairment because of predominant white matter injury. Also, strabismus increased in children born preterm with bilateral CP because of predominant white matter injury but with severe gross motor involvement. Conversely, basic visual functions were ameliorated in individuals born at term, with bilateral CP, and mild fine and gross motor involvement. Our data are in line with the literature reporting a poorer visual outcome in individuals born preterm and a better visual prognosis for those born at term.^49^, ^50^ This finding could be related to the timing, location, and extent of the brain injury, which could influence the process of neuronal plastic reorganization.^45^ Usually, a hypoxic insult could cause lesions to the periventricular white matter in infants born preterm; and to the grey matter (also involving the visual cortex), hippocampus, brainstem, and thalamic regions in infants born at term.^13^, ^51^ Damage to the optic radiation (located in the periventricular region) was predictive of minor visual recovery than injury to the occipital area.^11^, ^49^, ^52^ In addition, early damage occurring in an infant born preterm may prevent crucial tropic factors from being released and may result in more extensive visual pathway damage.^52^ Finally, our findings on significant visual improvement in children with mild motor impairments are in line with those by Matsuba and Jan^10^ who reported that children with fewer comorbid conditions (such as independent ambulation) were more likely to improve. The outcome of smooth pursuit and saccades was not related to peculiar clinical and instrumental features, while fixation improved mainly in children not assessed for CVD. As individuals who did not undergo the cognitive visual assessment had a more severe clinical profile, we can speculate that fixation improved significantly more in the children not assessed for CVD and less so in the others because they were already ‘stable’ at T0 in most cases. The small number of enrolled children and the lack of a detailed neuroradiological description could possibly explain these results.

This study has several potential limitations that should be considered. First, for methodological reasons, we chose to enrol only children with CP to characterize the natural history of CVI. Thus, generalizing our observations to all individuals affected by CVI (i.e. other associated causes) should be done with caution. Second, there is a need to evaluate oculomotor functions using more quantitative accuracy. Future studies should be carried out using more sophisticated quantitative assessments, such as eye‐tracking recordings, to objectively evaluate oculomotor functions. Finally, the current sample size of recruited participants may have limited the generalizability of the results.

In conclusion, characterizing the developmental trajectory of CVI is of pivotal importance to better understand its characteristics, improve the counselling offered to families, and define the type, timing, and efficacy of habilitative interventions, directing resources towards those functions that can still be improved and, at the same time, preventing them from being further compromised. A diagnosis of CVI was confirmed in the entire study sample and at each time point. However, except for a few ophthalmological and orthoptic problems that were stable over time, all other visual functions changed with age. Specifically, ocular fundus abnormalities and strabismus worsened, while oculomotor (fixation, smooth pursuit, and saccades) and basic visual (visual acuity, contrast sensitivity, and visual field) dysfunctions improved considerably, reaching complete recovery in some children at preschool and school ages. These data contribute to our further understanding of the natural history of CVI, emphasizing the ‘permanent’ but ‘not unchanging’ nature of this brain‐based visual impairment.

## FUNDING INFORMATION

The authors declare that the research was conducted in the absence of any commercial or financial relationships.

## CONFLICT OF INTEREST

The authors declare no conflicts of interest.

## Supporting information


**Table S1:** Neurovisual profiles at T2 in children who underwent the CVD assessment and those who did not perform the evaluation

**Table S2:** Prediction of the presence of a CVD at T2 by earlier vision problems

**Table S3:** Prediction of a visual motor impairment at T2 by earlier vision problems

**Table S4:** Prediction of a visual perceptual impairment at T2 by earlier vision problems

**Table S5:** Prediction of a widespread impairment at T2 by earlier vision problems

**Appendix S1:** Supplementary materials

## Data Availability

The raw data supporting the conclusions of this article will be made available by the authors, without undue reservation.
