# Supplementary material for: Natural history of cerebral visual impairment in children with cerebral palsy
Source: Dev Med Child Neurol. 2024 Sep 24;67(4):486–95. doi: 10.1111/dmcn.16096 (PMC11875525; doi:10.1111/dmcn.16096)
Supplement: Supplementary file 1 — Table S1: Neurovisual profiles at T2 in children who underwent the CVD assessment and those who did not perform the evaluation [file DMCN-67-486-s001.docx]

**Table S1- Neurovisual profiles at T2 in children who underwent the CVD assessment (subgroup 1, n=25) and those who did not perform the evaluation (subgroup 2, n=26).**

|  | **T2** | | |
| --- | --- | --- | --- |
|  | **Subgroup 1**  **N =25 (%)** | **Subgroup 2**  **N= 26 (%)** | **p-value** |
| **Refractive errors** | 24 (96) | 26 (100) | *p=0.49* |
| **Anterior Segment abnormalities** | 1 (4) | 3 (11) | *p=0.60* |
| **Ocular fundus abnormalities** | 14 (56) | 23 (88) | ***p=0.01*** |
| **Strabismus** | 19 (76) | 23 (88) | *p=0.29* |
| **Extrinsic Ocular Motility deficit** | 12 (48) | 15 (58) | *p=0.57* |
| **Nystagmus** | 5 (20) | 13 (50) | ***p=0.03*** |
| **Fixation abnormalities** | 1 (4) | 9 (35) | ***p=0.01*** |
| **Smooth pursuit abnormalities** | 7 (28) | 18 (69) | ***p<0.05*** |
| **Saccadic abnormalities** | 9 (36) | 23 (88) | ***p<0.05*** |
| **Visual acuity deficit** | 5 (20) | 23 (88) | ***p<0.05*** |
| **Altered contrast sensitivity** | 0 | 15 (58) | ***p<0.05*** |
| **Visual field limitation** | 1 (4) | 16 (61) | ***p<0.05*** |
| **IQ level** |  |  |  |
| Mean value ± SD (range) | 89.2 ± 20.6 (52-133) | Not evaluable (<70) |  |
| **GMFCS** |  |  |  |
| Mild (Level I-II) | 19 (76) | 5 (19) | ***p<0.05*** |
| Moderate (Level III) | 3 (12) | 2 (8) | *p=0.66* |
| Severe (Level IV-V) | 3 (12) | 19 (73) | ***p<0.05*** |
