# Supplementary material for: Natural history of cerebral visual impairment in children with cerebral palsy
Source: Dev Med Child Neurol. 2024 Sep 24;67(4):486–95. doi: 10.1111/dmcn.16096 (PMC11875525; doi:10.1111/dmcn.16096)
Supplement: Supplementary file 2 — Table S2: Prediction of the presence of a CVD at T2 by earlier vision problems [file DMCN-67-486-s002.docx]

**Table S2 - Prediction of the presence of a CVD at T2 by earlier vision problems (T0 and T1)**

|  | **Presence of CVD at T2** | |
| --- | --- | --- |
|  | **Odds Ratio (CI 95%); p-value** | |
|  | **T0** | **T1** |
| **Refractive errors** |  |  |
| Astigmatism | 0.00; *p>0.9* | 0.00; *p>0.9* |
| Hypermetropia | 0.46 (0.02; 3.96); *p=0.5* | 0.00; *p>0.9* |
| Myopia | 34.014.351 (0.00, NA); *p>0.9* | 44.480.305 (0.00, NA); *p>0.9* |
| **Anterior Segment abnormalities** |  |  |
| **Ocular fundus abnormalities** | 1.71 (0.20, 37.4); *p=0.7* | 3.27 (0.39, 70.3); *p=0.3* |
| **Strabismus** | 2.25 (0.31, 20.2); *p=0.4* | 6.00 (0.76, 59.7); *p=0.09* |
| Esotropia | 6.00 (0.72, 130); *p=0.14* | 9.33 (1.10, 205); *p=0.06* |
| Exotropia | 0.00; *p>0.9* | 0.44 (0.03, 10.9); *p=0.5* |
| **Extrinsic Ocular Motility deficit** | 1.23 (0.17, 10.9); *p=0.8* | 174,647,787 (0.00, NA); *p>0.9* |
| **Nystagmus** | 1.33 (0.15, 29.4); *p=0.8* | 1.33 (0.15, 29.4); *p=0.8* |
| **Fixation^a^ abnormalities** |  |  |
| Unstable | 1.54 (0.17, 34.1); *p=0.7* | 11.818.003 (0.00, NA); *p>0.9* |
| Not elicited | 13.090.711 (0.00, NA); *p>0.9* | - |
| **Smooth pursuit^b^ abnormalities** |  |  |
| Discontinuous | 12.0 (1.38, 267); ***p=0.04*** | 6.00 (0.72, 130); *p=0.14* |
| **Saccades^c^** |  |  |
| Saccadic amplitude abnormalities | 3.00 (0.40, 28.0); *p*=0.3 | 2.25 (0.31, 20.2); *p=0.4* |
| Saccadic latency abnormalities | 5.00 (0.59, 109); *p=0.2* | 4.00 (0.48, 85.9); *p=0.2* |
| **Visual acuity deficit** | 4.89 (0.59, 105); *p=0.2* | 1.33 (0.15, 29.4); *p=0.8* |
| **Altered contrast sensitivity** | 130.985.839 (0.00, NA); *p>0.9* | 9.454.403 (0.00, NA); *p>0.9* |
| **Visual field limitation** | 0.44 (0.05, 3.27); *p=0.4* | 1.00 (0.10, 22.5); *p>0.9* |
