# Supplementary material for: Natural history of cerebral visual impairment in children with cerebral palsy
Source: Dev Med Child Neurol. 2024 Sep 24;67(4):486–95. doi: 10.1111/dmcn.16096 (PMC11875525; doi:10.1111/dmcn.16096)
Supplement: Supplementary file 3 — Table S3: Prediction of a visual motor impairment at T2 by earlier vision problems [file DMCN-67-486-s006.docx]

**Table S3 - Prediction of a visual motor impairment at T2 by earlier vision problems (T0 and T1)**

|  | **Presence of visual perceptual impairment at T2** | |
| --- | --- | --- |
|  | **Odds Ratio (CI 95%); p-value** | |
|  | **T0** | **T1** |
| **Refractive errors** |  |  |
| Astigmatism | 154.198.393 (0.00, NA); *p>0.9* | 0.92 (0.03, 25.1); *p>0.9* |
| Hypermetropia | 0.89 (0.16, 4.94); *p=0.9* | 0.55 (0.06, 3.99); *p=0.6* |
| Myopia | 2.40 (0.20, 56.3); *p=0.5* | 3.93 (0.65, 33.2); *p=0.2* |
| **Anterior Segment abnormalities** |  |  |
| **Ocular fundus abnormalities** | 0.75 (0.12, 4.38); *p=0.7* | 0.58 (0.11, 2.92); *p=0.5* |
| **Strabismus** | 2.33 (0.47, 12.8); *p=0.3* | 3.13 (0.52, 26.2); *p=0.2* |
| Esotropia | 3.20 (0.65, 18.1); *p=0.2* | 3.50 (0.68, 21.9); *p=0.15* |
| Exotropia | 0.00; *p>0.9* | 0.50 (0.02, 5.98); *p=0.6* |
| **Extrinsic Ocular Motility deficit** | 0.83 (0.17, 4.10); *p=0.8* | 1.60 (0.33, 8.22); *p=0.6* |
| **Nystagmus** | 1.11 (0.17, 7.42); *p>0.9* | 1.11 (0.17, 7.42); *p>0.9* |
| **Fixation^a^ abnormalities** |  |  |
| Unstable | 9.17 (1.13, 199); *p=0.06* | 1.09 (0.04, 29.9); *p>0.9* |
| Not elicited | 1.83 (0.06, 52.3); *p=0.7* | - |
| **Smooth pursuit^b^ abnormalities** |  |  |
| Discontinuous | 5.83 (1.02, 48.9); *p=0.06* | 3.20 (0.65, 18.1); *p=0.2* |
| **Saccades^c^** |  |  |
| Saccadic amplitude abnormalities | 0.60 (0.10, 3.24); *p=0.6* | 0.63 (0.12, 3.06); *p=0.6* |
| Saccadic latency abnormalities | 3.50 (0.66, 21.6); *p=0.2* | 1.60 (0.33, 8.22); *p=0.6* |
| **Visual acuity deficit** | 2.24 (0.46, 11.9); *p=0.3* | 2.75 (0.43, 23.6); *p=0.3* |
| **Altered contrast sensitivity** | 2.38 (0.44, 15.0); *p=0.3* | 0.00; *p>0.9* |
| **Visual field limitation** | 0.43 (0.08, 2.11); *p=0.3* | 0.67 (0.08, 4.88); *p=0.7* |
