# Supplementary material for: Natural history of cerebral visual impairment in children with cerebral palsy
Source: Dev Med Child Neurol. 2024 Sep 24;67(4):486–95. doi: 10.1111/dmcn.16096 (PMC11875525; doi:10.1111/dmcn.16096)
Supplement: Supplementary file 4 — Table S4: Prediction of a visual perceptual impairment at T2 by earlier vision problems [file DMCN-67-486-s003.docx]

**Table S4 - Prediction of a visual perceptual impairment at T2 by earlier vision problems (T0 and T1)**

|  | **Presence of visual motor impairment at T2** | |
| --- | --- | --- |
|  | **Odds Ratio (CI 95%); p-value** | |
|  | **T0** | **T1** |
| **Refractive errors** |  |  |
| Astigmatism | 0.00; *p>0.9* | 2.29 (0.08, 63.7); *p=0.6* |
| Hypermetropia | 0.61 (0.07, 3.72); *p=0.6* | 0.00; *p>0.9* |
| Myopia | 24.311.321 (0.00, NA); *p>0.9* | 92.519.034 (0.00, NA); *p>0.9* |
| **Anterior Segment abnormalities** |  |  |
| **Ocular fundus abnormalities** | 1.25 (0.20, 10.6); *p=0.8* | 2.67 (0.46, 22.0); *p=0.3* |
| **Strabismus** | 1.43 (0.26, 8.08); *p=0.7* | 4.67 (0.75, 34.0); *p=0.11* |
| Esotropia | 2.38 (0.44, 15.0); *p=0.3* | 4.00 (0.71, 26.6); *p=0.13* |
| Exotropia | 0.00; *p>0.9* | 0.93 (0.08, 22.1); *p>0.9* |
| **Extrinsic Ocular Motility deficit** | 1.48 (0.27, 9.18); *p=0.7* | 10.0 (1.35, 210); ***p=0.05*** |
| **Nystagmus** | 0.92 (0.14, 8.01); *p>0.9* | 0.92 (0.14, 8.01); *p>0.9* |
| **Fixation^a^ abnormalities** |  |  |
| Unstable | 0.83 (0.12, 7.43); *p=0.9* | 22.690.567 (0.00, NA); *p>0.9* |
| Not elicited | 0.42 (0.01, 11.9); *p=0.6* | - |
| **Smooth pursuit^b^ abnormalities** |  |  |
| Discontinuous | 5.42 (0.93, 38.2); *p=0.06* | 5.50 (0.94, 46.5); *p=0.07* |
| **Saccades^c^** |  |  |
| Saccadic amplitude abnormalities | 4.58 (0.77, 32.7); *p=0.1* | 3.06 (0.55, 19.6); *p=0.2* |
| Saccadic latency abnormalities | 4.50 (0.75, 38.6); *p=0.12* | 10.0 (1.35, 210); ***p=0.05*** |
| **Visual acuity deficit** | 4.29 (0.74, 35.7); *p=0.13* | 0.92 (0.14, 8.01); *p>0.9* |
| **Altered contrast sensitivity** | 279.436.456 (0.00, NA); *p>0.9* | 7.303.968 (0.00, NA); *p>0.9* |
| **Visual field limitation** | 0.70 (0.12, 3.90); *p=0.7* | 2.15 (0.25, 46.6); *p=0.5* |
