# Supplementary material for: Natural history of cerebral visual impairment in children with cerebral palsy
Source: Dev Med Child Neurol. 2024 Sep 24;67(4):486–95. doi: 10.1111/dmcn.16096 (PMC11875525; doi:10.1111/dmcn.16096)
Supplement: Supplementary file 5 — Table S5: Prediction of a widespread impairment at T2 by earlier vision problems [file DMCN-67-486-s004.docx]

**Table S5 - Prediction of a widespread impairment at T2 by earlier vision problems (T0 and T1)**

|  | **Presence of widespread impairment at T2** | |
| --- | --- | --- |
|  | **Odds Ratio (CI 95%); p-value** | |
|  | **T0** | **T1** |
| **Refractive errors** |  |  |
| Astigmatism | 80.064.550 (0.00, NA); *p>0.9* | 25.526.887 (0.00, NA); *p>0.9* |
| Hypermetropia | 1.00 (0.19, 6.05); *p>0.9* | 0.57 (0.09, 3.64); *p=0.5* |
| Myopia | 2.29 (0.24, 22.5); *p=0.5* | 6.25 (1.09, 43.7); ***p=0.04*** |
| **Anterior Segment abnormalities** |  |  |
| **Ocular fundus abnormalities** | 0.45 (0.06, 2.55); *p=0.4* | 0.50 (0.08, 2.54); *p=0.4* |
| **Strabismus** | 1.60 (0.31, 9.53); *p=0.6* | 4.00 (0.53, 83.2); *p=0.2* |
| Esotropia | 2.00 (0.39, 11.9); *p=0.4* | 2.80 (0.50, 22.5); *p=0.3* |
| Exotropia | 0.00; *p>0.9* | 0.63 (0.03, 5.85); *p=0.7* |
| **Extrinsic Ocular Motility deficit** | 1.00 (0.19, 5.07); *p>0.9* | 1.96 (0.39, 10.6); *p=0.4* |
| **Nystagmus** | 0.57 (0.07, 3.33); *p=0.6* | 0.57 (0.07, 3.33); *p=0.6* |
| **Fixation^a^ abnormalities** |  |  |
| Unstable | 2.40 (0.42, 14.5); *p=0.3* | 0.63 (0.03, 5.85); *p=0.7* |
| Not elicited | 0.00; *p>0.9* | - |
| **Smooth pursuit^b^ abnormalities** |  |  |
| Discontinuous | 7.11 (0.98, 147); *p=0.09* | 4.37 (0.79, 35.3); *p=0.11* |
| **Saccades^c^** |  |  |
| Saccadic amplitude abnormalities | 1.00 (0.17, 6.33); *p>0.9* | 0.88 (0.17, 4.67); *p=0.9* |
| Saccadic latency abnormalities | 5.00 (0.84, 42.5); *p=0.1* | 3.67 (0.70, 23.0); *p=0.14* |
| **Visual acuity deficit** | 42.50 (0.49, 15.0); *p=0.3* | 1.30 (0.21, 7.31); *p=0.8* |
| **Altered contrast sensitivity** | 3.25 (0.62, 18.7); *p=0.2* | 0.00; *p>0.9* |
| **Visual field limitation** | 0.40 (0.07, 2.03); *p=0.3* | 0.74 (0.09, 4.53); *p=0.8* |
