# Supplementary material for: Natural history of cerebral visual impairment in children with cerebral palsy
Source: Dev Med Child Neurol. 2024 Sep 24;67(4):486–95. doi: 10.1111/dmcn.16096 (PMC11875525; doi:10.1111/dmcn.16096)
Supplement: Supplementary file 6 — Appendix S1: Supplementary materials [file DMCN-67-486-s005.docx]

**SUPPLEMENTARY MATERIALS - S1**

The neurovisual evaluation includes the assessment of oculomotor and basic visual functions as follows: Fixation was defined as present (score 1: stable for more than 3s), mildly impaired (score 2: unstable for less than 3 s), moderately impaired (score 3: difficult to evoke), or severely impaired (score 4: not elicited). Smooth pursuit was defined as present (score 1: continuous), mildly impaired (score 2: discontinuous), or severely impaired (score 3: not elicited). Saccadic movements were defined as present (score 1: both latency and amplitude were normal), normometric with increased latency (score 2: amplitude was normal), dysmetric but with normal latency (score 3: altered amplitude as multiple saccades were necessary to reach the target), dysmetric with increased latency (score 4: amplitude was altered and latency was increase), or absent (score 5). Concerning basic visual functions, binocular visual acuity was evaluated under full refractive correction using Teller Acuity Cards^1^, Lea Symbols, or letter optotypes^2^ according to the patient’s age and level of testing cooperation. Visual acuity values were expressed in cycles per degree (cyc/deg) or in tenths and were defined as “normal” or “reduced” using available normative data. Specifically, in children under 3 years of age, we applied normative data according to the Teller Acuity Cards handbook^1^. For patients aged between 3 and 5 years, we used age-specific norms according to the Current American Academy of Pediatrics guidelines^3^ (normal visual acuity >4 tenths for 36–47 months, >5 tenths for 48–59 months, and >6 tenths for ≥ 60 months of age). For subjects aged ≥ 6 years, we referred to the World Health Organization (WHO) International Classification of Disease-10 definition of visual impairment^4^ (normal vision > 8 tenths; deficient < 8 tenths). Contrast sensitivity was evaluated using the Hiding Heidi Low Contrast ‘‘Face” Test (HH). The ability to identify targets was considered as “normal” at 1.25% contrast level or as “reduced” when ≥ 2.5%.^5^ Finally, the ability to locate targets presented in different areas of the binocular visual field was evaluated using kinetic perimetry^6^, based on the main behavioral reactions of the subject (e.g., movements of the head, eyes, or a limb toward the target). We classified the visual field as “normal” or “reduced” according to age-specific normative data.^6-8^

Cognitive visual functions were evaluated using a battery of tests referring to visual motor and visual perceptual skills. Visual motor skills were analysed using the Developmental Test of Visual-Motor Integration -VMI-^9^ which is a paper-and-pencil test for visual motor integration abilities, and the Block Construction -BC- task which is a subtest of NEPSY, a Developmental Neuropsychological Assessment battery^10^ to assess constructional praxia. Visual perceptual skills were assessed using the Bova et al. (2007) battery^11^ that includes the evaluation of (1) perceptual categorization, meaning the ability to recognize the structural identity of an object when its projection on the retina is altered (using the Street Completion Test - Street, 1931^12^ - and the coloured photographs of objects viewed from unusual perspectives and~~,~~ photographs illuminated in unusual ways, newly developed tests created *ad hoc* by consulting a dictionary of selected objects from among those that recur most frequently in the vocabulary of school children), (2) the constancy of internal representation of objects (using a series of Imaginary Figures selected from the Object Decision test of the Birmingham Object Recognition Battery)^13^, and (3) semantic categorization, which is the capacity of recognizing semantic and functional attributes of stimuli (using two Matching Tasks in which the children were required to match pairs of drawings by class and by function; also in this case, items were selected from the Birmingham Object Recognition Battery)^13^. For each of the above-mentioned visual motor and visual perceptual tests, we calculated the *z* score. Performance was considered abnormal if the *z* score derived from normal controls was under -2. A visual motor or visual perceptual dysfunction was present if at least one of the tasks evaluating respectively these two skills was impaired. Finally, a cognitive visual disorder was considered present in the case of visual motor and/or visual perceptual dysfunction.

Neurovisual and cognitive-visual evaluations were carried out in two different sessions, lasting three hours. A multidisciplinary team performed the neurovisual evaluation. This included a child neuropsychiatrist and child therapist trained in neurovisual function evaluation of oculomotor/basic visual functions assessment. An ophthalmologist performed the ophthalmology evaluation and an orthoptist assessed the presence of strabismus, ocular motility deficits, and abnormal eye movements. Each evaluation was videotaped, allowing the team to observe and judge the child’s visual performance. In particular, qualitative functions such as fixation, smooth pursuit, and saccadic movements. Clinicians assessing the video were blinded to the overall purpose of the study as well as the aetiology and severity of CP.

1. Teller DY, McDonald MA, Preston K, Sebris SL, Dobson V. Assessment of visual acuity in infants and children: the acuity card procedure. *Dev Med Child Neurol.* 1986;28:779–789.
2. Hyvärinen L, Näsänen R, Laurinen P. New visual acuity test for preschool children. *Acta Ophthalmol.* 1980;58:507–511.
3. Donahue SP, Baker CN; Committee on Practice and Ambulatory Medicine, American Academy of Pediatrics; Procedures for the Evaluation of the Visual System by Pediatricians. *Pediatrics.* 2016;137(1):10.1542/peds.2015-3597.
4. World Health Organization [WHO] ICD-10: International Statistical Classification of Diseases and Related Health Problems: Tenth Revision, 2nd Edn. Geneva: World Health Organization, 2021.
5. Leat SJ, Wegmann D. Clinical testing of contrast sensitivity in children: age-related norms and validity. *Optom Vis Sci.* 2004;81(4):245-254.
6. van Hof-van Duin J, Heersema DJ, Groenendaal F, Baerts W, Fetter WP. Visual field and grating acuity development in low-risk preterm infants during the first 2 1/2 years after term. *Behav Brain Res.* 1992;49:115–122.
7. Heersma DJ, van-Hof-Van Duin J, Hop WCJ. Age norms for visual field development in children aged 0 to 4 years using arc perimetry. *Invest Ophthalmol Vis Sci.* 1989;30:242
8. Wilson M, Quinn G, Dobson V, Breton M. Normative values for visual fields in 4- to 12-year-old children using kinetic perimetry. *J Pediatr Ophthalmol Strabismus* 1991;28:151–154.
9. Beery KE, Buktenica NA. VMI Developmental Test Of Visual-Motor Integration, ed. C. Preda (Firenze: Giunti Os), 2000.
10. Korkman M, Kirk U, Kemp S. NEPSY, 2nd Edn. Firenze: Giunti OS, 2011.
11. Bova SM, Fazzi E, Giovenzana A, Montomoli C, Signorini SG, Zoppello M, et al. The development of visual object recognition in school-age children. *Dev Neuropsychol.* 2007;31:79–102.
12. Street RF. *A gestalt completion contribution to education*, New York: Columbia University, Teachers College, Bureau of Publication, 1931.
13. Riddoch, M. J., & Humphreys, G. W. (1993). Birmingham Object Recognition Battery. Hove, UK: Lawrence Erlbaum Associates, Inc
